# Cost of dengue in Colombia: A systematic review

**DOI:** 10.1371/journal.pntd.0012718

**Published:** 2024-12-12

**Authors:** Alfonso J. Rodríguez-Morales, Eduardo López-Medina, Iván Arboleda, Jaime A. Cardona-Ospina, Jaime Castellanos, Álvaro A. Faccini-Martínez, Elaine Gallagher, Riona Hanley, Pio López, Salim Mattar, Carlos Eduardo Pérez, Randee Kastner, Humberto Reynales, Fernando Rosso, Jing Shen, Wilmer E. Villamil-Gómez, Marcela Fuquen

**Affiliations:** 1 Grupo de Investigación Biomedicina, Faculty of Medicine, Fundación Universitaria Autónoma de las Américas-Institución Universitaria Visión de las Américas, Pereira, Risaralda, Colombia; 2 Master of Clinical Epidemiology and Biostatistics, Universidad Científica del Sur, Lima, Perú; 3 Colombian Association of Infectious Diseases (ACIN), Bogotá, D.C., Colombia; 4 Department of Pediatrics, Universidad del Valle, Cali, Valle del Cauca, Colombia; 5 Centro de Estudios en Infectología Pediátrica, Cali, Valle del Cauca, Colombia; 6 Clínica Imbanaco, Grupo Quirón Salud, Cali, Valle del Cauca, Colombia; 7 Baxalta Colombia SAS, (Takeda), Bogotá, D.C., Colombia; 8 Grupo de Investigación en Infecciones Emergentes y Medicina Tropical, Instituto para la Investigación en Ciencias Biomédicas, SCI-HELP, Pereira, Risaralda, Colombia; 9 Division of Infectious Diseases and Vaccinology, School of Public Health, University of California, Berkeley, California, United States of America; 10 Grupo de Virología, Vicerrectoría de Investigación, Universidad El Bosque, Bogotá D.C., Colombia; 11 Servicio de Infectología, Hospital Militar Central, Bogotá D.C., Colombia; 12 Servicios y Asesorías en Infectología, Bogotá D.C., Colombia; 13 Facultad de Medicina, Universidad Militar Nueva Granada, Bogotá, D.C., Colombia; 14 Takeda Pharmaceuticals International AG, Zurich, Switzerland; 15 Instituto de Investigaciones Biológicas del Trópico, Universidad de Córdoba, Montería, Córdoba, Colombia; 16 Centro de Atención e Investigación Médica—CAIMED, Chía, Cundinamarca, Colombia; 17 Infectious Diseases Service, Fundación Valle del Lili, Cali, Valle del Cauca, Colombia; 18 Facultad de Ciencias de la Salud, Universidad Icesi, Cali, Valle del Cauca, Colombia; 19 Centro de investigación en ciencias de la vida, Universidad Simón Bolívar, Barranquilla, Atlántico, Colombia; University of Heidelberg, GERMANY

## Abstract

**Background:**

Dengue is hyperendemic in Colombia. It imposes a substantial economic burden on patients, caregivers, society, and the national health system. We intend to identify and synthesize the evidence regarding the economic burden of dengue in Colombia.

**Methods:**

A systematic review (PROSPERO CRD42021257985) of economic studies was performed. A comprehensive search was completed in PubMed, EMBASE, the Cochrane Library, the LILACS, and SciELO databases. Study selection and data extraction was made by two researchers.

**Results:**

160 records were identified. Of these, 14 studies were selected for data extraction. Direct medical cost of dengue is mainly represented by hospitalization (USD 823 to 1,754). The annual aggregated cost is near to USD 159.6 million, with ambulatory care (USD 90.1 million) and fatal cases (USD 30.7 million) representing 75% of the total cost. The aggregate indirect cost (due to loss in income while sick or as a caretaker) was USD 92.8 million. Vaccination seems to reduce the economic cost of dengue.

**Conclusions:**

Dengue financial burden could be challenging for low-income communities as those affected in Colombia. An integrated approach including vector control and the introduction of a vaccine for dengue has the potential to reduce the economic burden of the disease.

## Author summary

Dengue fever is a significant public health concern globally. Colombia, like many other tropical and subtropical countries, faces the challenge of managing and mitigating the burden of dengue. We developed a systematic review of the costs of dengue in Colombia, and we believe our findings have the potential to inform public health policies and resource allocation. By analyzing the direct medical costs associated with dengue cases, including hospitalization, diagnostics, and treatment, policymakers can gain valuable insights into the resource requirements for managing and preventing the disease. We believe this information could be taken as an example for other countries with similar epidemiology and could help to prioritize targeted interventions to reduce the incidence of dengue, such as mosquito control programs, community awareness campaigns, and vaccination strategies. We concluded that: Dengue disproportionately affects the communities with low socioeconomic status, costs related to severe dengue are significantly higher than ambulatory cases, dengue leads to missed work and lost earnings due to death or disability, implementing vector control programs to combat dengue is costly, and introducing a dengue vaccine has the potential to reduce the economic burden.

## Introduction

Dengue is the most common arboviral disease around the world. It is estimated that 100 to 400 million infections occur yearly. The global incidence of dengue has grown over time, with an increase of 85.47% from 1990 to 2019. The burden of this disease is highest in regions with low or medium socio-demographic index (SDI) [1]. In the Americas, approximately 500 million people are at risk of dengue. In this region, dengue incidence has dramatically increased from 1.5 million cumulative cases reported in the 1980s to 16.2 million cases reported in the in the decade 2010–2019 [2].

Colombia is one of the most affected countries in the Americas. All four dengue virus serotypes (DENV1-4) circulate in the country, with epidemics occurring every 3 to 4 years. According to local data, dengue is hyperendemic in most regions of Colombia. As there is no seasonality in the country, cases are observed throughout the year with variable peak periods [3]. The national incidence of dengue for 2021 was 172.9 cases per 100,000 inhabitants at risk, with some regions with an incidence as high as greater than 400 cases per 100,000 inhabitants at risk. Approximately, 51.6% of dengue reported cases had warning signs, and 2.1% were classified as severe dengue (SD). However, in some regions, the proportion of SD is around 10%. Furthermore, 83% of dengue with warning signs (DWS) cases, and 95% of SD cases required hospitalization [4]. It is important to consider that underreporting of dengue cases in the country has been documented, related to several factors such as home management of mild cases and non-reporting of suspected cases by health professionals [5].

There is currently no specific treatment for dengue infection. Governmental prevention policies for dengue focus on active and reactive vector control, personal protection from mosquito bites, community engagement, education, and surveillance. The limitations of vector control, together with other factors such as urbanization and population growth in endemic regions, the continued geographic expansion of vectors in response to climate change, and increased domestic travel, suggest a continued increase in the incidence of dengue in the coming years which impose a significant challenge to the national public health [6].

Dengue causes a significant burden for patients, caregivers, and society. It is also associated with substantial and growing economic costs, including direct medical, non-medical and indirect costs. The problem is compounded by the fact that the same vectors transmit Zika, chikungunya, and yellow fever, further impacting an already struggling healthcare system [7]. The global economic and societal costs of dengue have been estimated to be approximately US$39 billion per year, with the Americas contributing up to US$4 billion annually. This high economic burden and the size of the at-risk population, confirm the global importance of dengue infections [8].

Although, dengue is a significant public health problem in Colombia, its impact has not been fully elucidated. Understanding the economic burden of this disease is critical to inform evidence-based health policy and prioritize preventive and control strategies. Therefore, we systematically reviewed the literature to identify and synthesize the existing evidence on the economic burden of dengue in Colombia.

## Methods

### Search strategy, eligibility criteria, and study selection

This systematic literature review was registered to PROSPERO (CRD42021257985), and it was conducted in line with the Cochrane Handbook for Systematic Reviews and the Preferred Reporting Items for Systematic Reviews and Meta-Analyses (PRISMA)(S1 PRISMA Checklist). A comprehensive search for the economic burden of dengue in Colombia was completed in the electronic databases PubMed, EMBASE, the Cochrane Library, Latin American and Caribbean Health Sciences Database (LILACS), and Scientific Electronic Library Online (SciELO). As a search strategy, we used (’dengue’ OR ’severe dengue’ OR ’dengue virus’ OR ’dengue hemorrhagic fever’) combined with terms related to costs and economic burden. We restricted our search to articles in English or Spanish published between 2010 to 2020. In addition, a specific search for grey literature was conducted on the websites of governmental and public health organizations, conferences, and main universities in Colombia (S1 Text and S1 Table).

We included economic evaluations and studies on the cost of illness and the economic burden of dengue, with information about costs to patients and health services (direct medical costs, direct non-medical costs, or indirect social costs), vector control and surveillance costs, and productivity loss. We excluded publications that did not clearly outline methods and sources for data collection or analysis, as well as news and opinion articles, case reports, narrative reviews, and letters.

The selection of studies was carried out in two phases. In the first phase, the title and abstract of the retrieved articles were independently screened by two reviewers against the eligibility criteria. Discrepancies between the reviewers were discussed, and if not resolved, a third reviewer made the final decision. In phase two, the full text of all articles retained in phase one was assessed further for eligibility. All citations found during the searches were stored in a reference database. In Microsoft Excel, economic data were collected in separate data extraction forms (DEFs).

### Quality assessment

We assessed the methodological quality of peer-reviewed publications only. The quality of the included studies were evaluated using the National Health Service (NHS) Wales tool. Based on the assessment of each question on the checklist, a total rating of good (>70% score), fair (50%-70% score), or poor (<50% score) quality was assigned to each study.

### Data extraction and synthesis of results

A descriptive summary of the extracted economic data was conducted. For studies reporting similar results, the most recent publication was considered. For the financial burden, all costs were converted to 2020 USD using the Colombia CPI (consumer price index) where possible.

## Results

### Study selection

**Fig 1:**
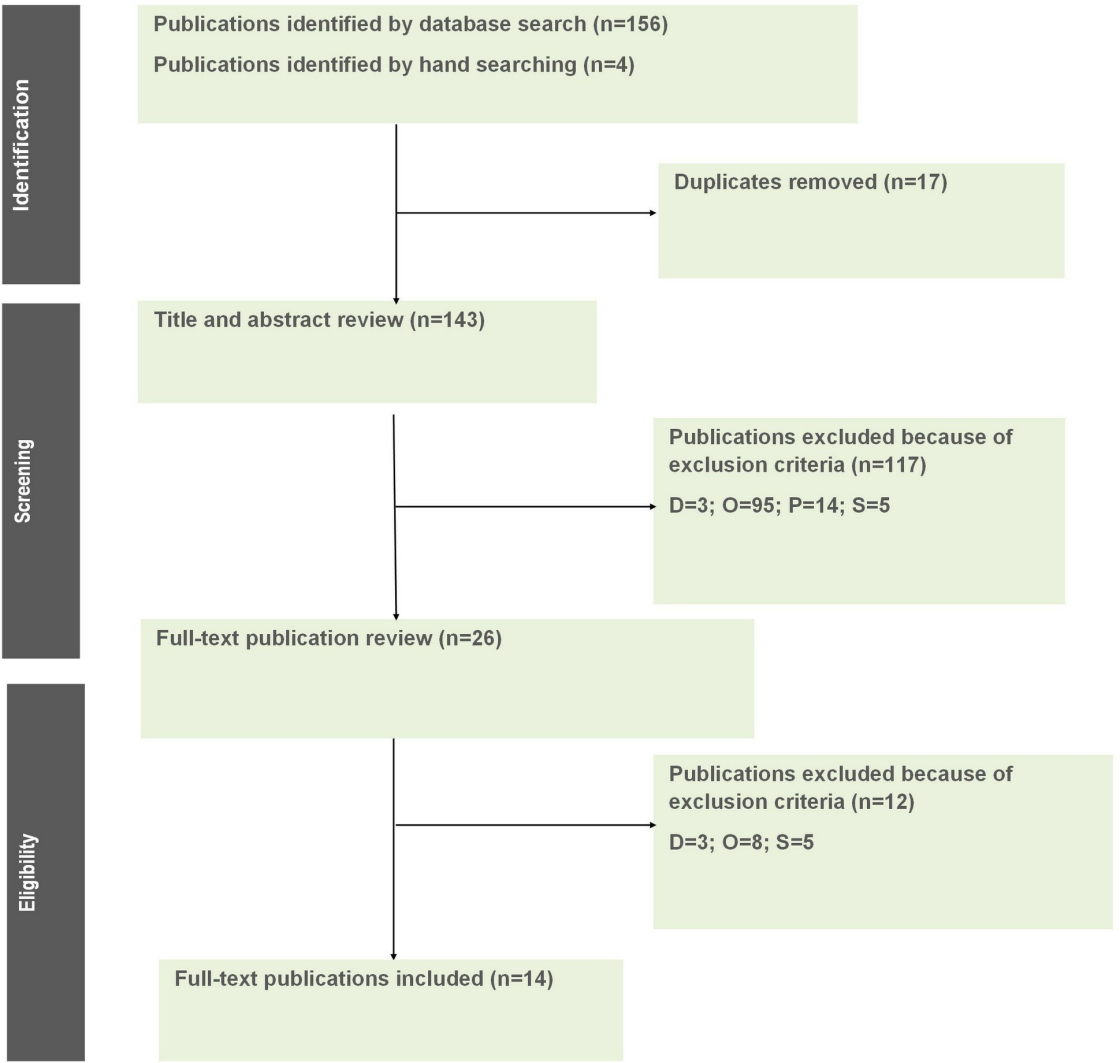
PRISMA diagram for economic studies. D, duplicates; O, outcome; P, population; S, study design.

A total of 156 records were identified from the literature search, and four publications through hand searching. After removing duplicate records, titles and abstracts of the remaining 143 publications were screened. 117 were excluded due to duplicated information (n = 3), outcomes (n = 95), population (n = 14), and study design (n = 5). Thus, 26 publications were selected for full-text review. After assessing the full text of these publications, 14 studies were selected for data extraction and inclusion in this SLR. The literature search results are presented in the PRISMA diagram below (Fig 1). The lists of the excluded publications at both stages of the review process are available in supplementary material (S2 Text).

### Risk of bias assessment

The quality of the 14 identified studies was assessed using the NHS Wales tool. Overall, seven studies were rated good, six were rated fair, and one was classified as poor quality. The full details of the risk of bias assessment are provided in the supplementary material (S2 Table).

### Characteristics of included economic burden studies

Table 1 summarizes the characteristics of the studies included. Nine studies presented national data [9–17] and five presented regional data [18–22]. Three studies were conducted from the societal perspective [9,13,15], three from the healthcare system perspective [10,11,16], three from the policy maker / service perspective [20–22], and two from the patient´s perspective [18,19]. Two studies did not report the perspective [12,14]. One study, a cost-effectiveness analysis of dengue vaccination, used both healthcare system and societal perspective [17]. Direct medical costs were not reported by four studies [12,14,20,22], three of which focused on vector control costs [12,20,22] and one was an ecological study that estimated the burden of dengue in terms of disability-adjusted life years (DALYs) [14]. Seven studies reported the indirect costs related to dengue [9,13,15–17,19,21]. Costs of ambulatory care and hospitalization were reported by nine studies [9–11,13,15–18,21].

**Table 1:** Characteristics of cost studies included in the systematic review.

Author, year	Mora-Salamanca, 2020 (14)	Vásquez-Trujillo, 2020 (18)	Claypool, 2019 (12)	Hernández-Sarmiento, 2019 (19)	Salinas-López, 2018 (22)	Zeng, 2018 (17)	El-Fezzazi, 2017 (13)	Fitzpatrick, 2017 (11)	Lee, 2017 (21)	Alfonso-Sierra, 2016 (20)	Shepard, 2016 (9)	Castro -Rodríguez, 2016 (15)	Castro -Rodríguez, 2015 (16)	Castañeda-Orjuela, 2012 (10)
**Study design**	Ecological exploratory study	Longitudinal study	Economic evaluation	Prospective, cross-sectional	Cost analysis	Cost-effectiveness analysis	Economic evaluation + RCT	Economic evaluation	Economic evaluation	Cost analysis + RCT	Systematic analysis	Burden of illness	Cost analysis	Economic evaluation
**Location**	National	Meta	National	Medellín and Monteria	Giron & Buga	National	National	National	Piedecuesta	Girardot	National	National	National	National
**Perspective**	NA	Longitudinal	NR	Patient	Policy maker	Healthcare system and societal	Societal	Healthcare system	Decision maker	Service provider, vector control program	Societal	Societal	Healthcare system and patients	Healthcare system
**Year of valuation and currency**	NA	NR; USD	2015; USD	NR; Colombian Pesos	2016; USD	2015; USD	2014; USD	2013; USD	2014; USD	2013; USD	2013; USD	2012; USD	2012; USD	2011; USD
**Direct Medical**	N	Y	N	Y	N	Y	Y	Y	Y	N	Y	Y	Y	Y
**Direct Non-medical**	N	N	N	N	N	N	Y	N	Y	N	Y	Y	Y	N
**Total direct**	N	N	N	N	N	Y	Y	N	N	N	Y	Y	N	N
**Indirect costs**	N	N	N	Y	N	Y	Y	N	Y	N	Y	Y	Y	N
**Vector control**	N	N	Y	N	Y	Y	N	Y	N	Y	N	Y	N	Y
**Surveillance**	N	N	N	N	N	N	N	N	N	N	N	Y	N	N
Overall*	N	N	N	N	Y	Y	N	N	Y	N	Y	Y	Y	Y

NA, not applicable; N, non; NR, not reported; RCT, randomized control trial; USD, United States dollar; Y: yes

*Total reported costs (direct, indirect, vector control and/or surveillance costs)

### National costs associated with dengue

Eight studies reported the costs of dengue at the national level [9–13,15–17]. Shepard et al. [9] estimated the economic burden of dengue in Colombia using dengue incidence estimates from Institute for Health Metrics and Global Burden of Disease Evaluation Study 2013. They reported a direct cost of USD 823 for hospitalized cases vs USD 135 and USD 20 for ambulatory and non-medical cases (ie, who received neither diagnosis nor treatment from a health professional or facility), respectively. The indirect non-medical cost was substantially higher than the direct non-medical cost (USD 118 vs USD 20, respectively). The cost of dengue deaths in children (using the human capital approach) was higher than that of adults (USD 300,047 vs USD 195,163 per case, respectively). The overall average cost of dengue per case was USD 313. The total annual aggregated cost was reported as USD 159.6 million, with ambulatory care (USD 90.1 million) and fatal cases (USD 30.7 million) representing 75% of the total cost. The aggregate indirect cost (due to loss in income while sick or as a caretaker) was USD 92.8 million [9].

Castañeda-Orjuela et al. [10] estimated the direct medical and indirect costs incurred due to dengue in Colombia from 2011 to 2014. The average cost per dengue case for outpatient ranged from USD 106 in 2011 to USD 120 in 2014. The average costs per dengue case for inpatient and SD were USD 1,108 and USD 1,754, respectively. Similar trends were observed for the previous years of the study period. The total medical costs for all dengue cases in an average year ranged from USD 16.1 million to 23.6 million. The authors also estimated the costs associated with the vector control program which ranged from USD 51.8 million to USD 59.3 million, showing a high economic burden of dengue from the vector control program by the government/community [10]

Castro-Rodríguez et al. [15] used information from official databases and face-to-face surveys to estimate the financial burden of dengue for households in Colombia. From 2010 to 2012, the average costs per case for ambulatory cases, hospitalized cases of DWS, and hospitalized cases of SD were USD 72, USD 319, and USD 2048, respectively. The average direct medical costs to the households for hospitalized cases of DWS and hospitalized cases of SD were USD 47 and USD 78, respectively. Direct non-medical costs (transport, caregivers’ fee, lodging, food, and post-disease expenses) for both cases were also high at USD 63 and USD 85, respectively. In contrast, the cost for ambulatory cases was USD 40. Overall, indirect costs (financial costs due to loss of workdays/schooldays) contributed the highest cost burden to the households, with USD 151, USD 256, and USD 274 reported for ambulatory cases, hospitalized DWS, and SD, respectively [15].

Castro-Rodríguez et al. [16] further analyzed the data collected (described above) and estimated the costs to the health system and total costs for households in Colombia. In 2010 (an epidemic year), the total cost to the health system was USD 39.2 million, whereas in 2011 and 2012 (endemic years), the healthcare costs were USD 11.9 million and USD 13.1 million, respectively. They further calculated the lost income due to premature dengue deaths. The lost incomes were USD 23.9, USD 3.9, and USD 3.9 million for 2010, 2011, and 2012, respectively. The total costs to the households (sum of direct medical costs, direct non-medical costs, indirect costs, and lost income due to death) for 2010, 2011, and 2012 were USD 86, USD 20.8, and USD 25.8 million, respectively [16]. Further details on direct and indirect costs reported in seven studies are summarized in Table 2.

**Table 2:** National direct and indirect costs of dengue in Colombia.

Author, year	Setting	Costs		
Shepard et al., 2016 [9]	Average cost per dengue case by treatment setting			
		Direct cost per non-fatal case	Indirect cost per non-fatal case	Dengue deaths cost/case
	Hospital cases	$823	$211	NA
	Ambulatory cases	$135	$118	NA
	Non-medical cases	$20	$118	NA
	Child	NA	NA	$300,047
	Adults	NA	NA	$195,163
	Aggregate cost by dengue cases in a year			
		Direct costs	Indirect costs	Total
	Hospital cases	$15,908,942	$4,081,084	$19,990,026
	Ambulatory cases	$48,077,875	$42,020,164	$90,098,039
	Non-medical cases	$2,790,097	$16,056,836	$18,846,933
	Fatal cases	NA	$30,658,066	$30,658,066
	Aggregate costs	$66,776,914	$92,816,150	$159,593,064
	Cost per case of dengue	$313		
Castañeda-Orjuela et al. 2012 [10]	Average cost per dengue case by treatment setting (2014)			
	Outpatient case	$120		
	Inpatient case	$1,108		
	Severe dengue case	$1,754		
	Aggregate costs of all dengue cases (2014)			
	Outpatient cases	$1,242,393		
	Inpatient cases	$17,261,112		
	Severe dengue cases	$1,601,415		
	Total costs	$20,104,920		
	Vector control cost			
	Average annual cost per inhabitant (department)	$7,638		
	Average annual cost per inhabitant (municipality)	$1,143		
	Total average annual cost	$ 518,112,667–59,262,029		
El Fezzazi et al. 2017 [13]	Average costs per participant			
	Control Group (Non–vaccinated)			
	Hospitalization	$3.37		
	Consultation	$4.41		
	Absence costs	$0.25		
	Travel costs	$4.28		
	Total costs	$12.31		
	Vaccinated group			
	Hospitalization	$0.55		
	Consultation	$1.46		
	Absence costs	$0.08		
	Travel costs	$1.05		
	Total costs	$3.14		
Castro-Rodríguez et al. 2015 [16]	Average costs per case (2012)			
	Ambulatory DF	$64		
	Hospitalized DF	$308		
	DHF	$2,688		
	Average costs per household, Ambulatory DF (2012)			
	Direct medical	$18		
	Direct non-medical	$40		
	Indirect	$151		
	Total	$210		
	Average costs per household, hospitalized DF (2012)			
	Direct medical	$47		
	Direct non-medical	$63		
	Indirect	$256		
	Total	$367		
	Average costs per household, DHF (2012)			
	Direct medical	$78		
	Direct non-medical	$85		
	Indirect	$274		
	Total	$437		
Castro-Rodríguez et al. 2016 [15]	Total costs (2012)			
	Cost to health system	$13,052,793		
	Cost to households			
	Direct medical	$1,780,815		
	Direct non-medical	$2,889,602		
	Indirect	$4,126,594		
	Loss of income due to death	$3,939,625		
	Total	$25,789,430		
Zeng et al. 2018 [17]	Average costs per case			
	Hospitalized	$589		
	Ambulatory	$74		
	Vaccine delivery	$4		
	Indirect (hospitalized)	$154		
	Indirect (ambulatory)	$86		
	Vaccine dose	$3		
	Cost per capita			
	Non-vaccinated disease	$6		
	Difference in disease cost (routine 1 vaccination)	-$2		
	Difference in disease (1 +follow up after 4 years)	-$2		
	Difference in disease (1+ follow up after 8 years)	-$2		
Fitzpatrick et al. 2017 [11]	Ambulatory clinic visit	$65 (95% CI $13-$189)		
	Hospital bed day, primary	$257 (95% CI $101-$538)		
	Hospital bed, day. Specialist	$310 (95% CI $113-$666)		
	Medical case management only	$99 (95% CI $30-$311)		

CI, confidence interval; NA, not applicable; DF, dengue fever; DHF, dengue hemorrhagic fever

Two studies reported economic data in the scenario of the introduction of a new dengue vaccine. El Fezzazi et al. [13] evaluated the direct and indirect medical costs associated with dengue cases after introducing a new dengue vaccine among children 9 to 16 years old within the context of a phase III clinical trial. Participants were followed for 25 months where virological confirmed dengue (VCD) cases were registered. Cost data of each VCD case including hospitalization, consultation, travel, and absence costs were analyzed comparing the vaccinated and control groups. Overall, the average costs per dengue episode were higher in the non-vaccinated group than in the vaccinated group. Hospitalization and consultation costs were USD 3.7 and USD 0.55 in the vaccinated group and USD 4.41 and USD 1.46 in the non-vaccinated group, respectively. The total cost of dengue per patient dropped from USD 12.3 in the control group to USD 3.13 in the vaccinated group. However, it is important to note that vaccination costs were not included in the cost calculations [13].

Zeng et al. analyzed the cost-effectiveness of dengue vaccination. Their results were based on phase III trial data from 10 dengue endemic countries (including Colombia) and mathematical model predictions covering a 30-year span and assuming a vaccine coverage of 80% (first dose). Cost was estimated from the health system´s perspective including costs of vaccine purchase and delivery (20 USD per dose, total of 3 doses) and treatment of dengue; the societal perspective included indirect costs of illness and premature death, and opportunity costs of time required to obtain each dengue dose. Effectiveness was calculated as a reduction of the incidence of symptomatic dengue episodes over 30 years by an average of 23.1%. For Colombia, the total hospitalization and ambulatory care costs per case were USD 589 and USD 74, respectively. The indirect cost per case was USD 154 for hospitalized patients and USD 86 for ambulatory patients. The decrease in annual costs with the vaccine introduction (80% coverage) in the 9 to 16 years old group ranged from USD—1.5 to– 1.8 per capita compared to no vaccination. From the perspective of the Colombian health system, routine dengue vaccination would be at least cost-effective, with an incremental cost-effectiveness ratio of USD 4,906/DALY (CI 95% 2,560–8,481) averted with R9 (routine vaccination at age 9 only), USD 5,134/DALY (CI 95% 2,718–8,786) with R9C4 (routine vaccination at age 9 plus 4 catch-up cohorts [ages 9–13]) and USD 5,499/DALY (CI 95% 3,015–9,261) with R9C8 (routine vaccination at age 9 plus 8 catch-up cohorts [ages 9–17]). From a societal perspective, routine dengue vaccination is cost-saving with USD 518/DALY (CI 95% −2,808–5,412), USD 775/DALY (CI 95% −2,588–5,634), and USD 1,184/DALY (CI 95% −2,297–6,086) with R9, R9C4 and R9C8, respectively [17].

### Regional costs associated with dengue

Five studies reported the costs of dengue at the regional level [18–22]. All costs were converted to 2020 USD, except for two studies that did not report the costing year [18,19]. The results of the studies are summarized below.

Vasquez-Trujillo et al. evaluated the costs associated with dengue in a hyperendemic region between 2010 and 2016. The cost per case during this period ranged from USD 196 to USD 509, with hospitalization as the main cost driver. Overall, the costs were proportionately higher in the epidemic years of 2010, 2013 and 2014. Also, these years were associated with increased mortality and a higher proportion of years lost due to death. Between 2014 and 2016, the estimated costs of years lost due to disability were substantially higher for women because more cases were observed in female children below the age of 15 years [18].

Hernandez-Sarmiento et al. surveyed the population of two Colombian cities (Medellin and Monteria) for out-of-pocket expenses incurred during the diagnosis and recovery from dengue. Out of pocket expenses represented between 9% and 45% of the income, and transport to the care center was the main cost driver. Few had out-of-pocket expenses for medications [19].

Lee et al. [21] estimated the economic burden of dengue from diagnosis to recovery in a northern city in Colombia in 2014. Overall, the average total costs per dengue episode were higher for inpatient than outpatient cases, including: direct medical (USD 316 vs USD 43), non-medical (USD 35 vs USD 22), and indirect costs (USD 146 vs USD 138). All expenses were higher in adults (> = 15 years) than in children (<15 years), except for direct non-medical costs (USD 30 in adults vs USD 43 in children) [21].

Two studies informed the costs associated with vector control programs. Alfonso-Sierra et al. reported a total cost of 29 USD per house in a high-incidence area [20]. Salinas-Lopez et al. reported an annual total cost of vector control programs of USD 167,627 in a city with 180,377 inhabitants and USD 126,362 in a city with 115,026 inhabitants [22].

### Societal impact (productive days lost)

Three studies examined the societal impact of dengue in Colombia, but the data were limited. Lee et al. reported 5.8 partial and 3.1 full days of productivity loss for inpatients and 4.7 partial and 2.8 full days of productivity loss for outpatients [21]. According to severity, Rodriquez et al. reported a loss of 14.41 productive days for SD and 8.32 to 13 days of productivity loss for ambulatory cases [16]. Finally, El Fezzazi et al. reported the productivity days lost due to dengue in vaccinated versus non-vaccinated patients in Colombia, using phase III clinical trial data. Overall, the vaccinated group lost 0.4 productive days, while the non-vaccinated group lost 0.9 productive days. In both groups, hospitalized cases lost more work/school days than non-hospitalized cases due to the severity of their illness [13]. Further details on productive days lost due to dengue are summarized in Table 3.

**Table 3:** Productive days lost due to dengue.

Author, year	Region	Costing year	Setting	Number of full days lost	Number of partial days lost
Lee et al. 2017 [21]	Piedecuesta	2014	Inpatient	5.8 (2.2)	3.1 (1.5)
			Outpatient	4.7 (2.0)	2.1 (1.9)
EL-Fezzazi et al. 2017 [13]	Nationwide	2017	VCD (all hospitalized cases)	Work:2.9 School:6.8	NR
			VCD (hospitalized vaccinated)	Work: 2 School:7	
			VCD- (hospitalized non-vaccinated)	Work:3.1 School:6.7	
			VCD (all non-hospitalized cases)	Work:0.4 School:2.8	
			VCD (non-hospitalized vaccinated)	Work:0.3 School:2.7	
			VCD (non-hospitalized non-vaccinated)	Work:0.4 School:2.9	
			VCD (all cases)	Work: 0.7 School:3.3	
			VCD (all vaccinated)	Work:0.4 School:2.9	
			VCD (all non-vaccinated)	Work:0.9 School:3.5	
Castro-Rodríguez et al. 2015 [16]	Nationwide	2012	Inpatient DF	8,32	NR
			Outpatient DF	13	
			DHF	14.41	

DF, dengue fever; DHF, dengue hemorrhagic fever; NR, not reported; VCD, virologically confirmed dengue

## Discussion

The economic and societal burden of dengue in Colombia is considerable, as it affects the health and well-being of millions of people, particularly in endemic regions. In addition to the disease’s physical effects, dengue also significantly impacts productivity and income. People affected may have to take time off work or school, resulting in a loss of income and increased financial stress for families. This situation can have a cascading effect on communities, as reduced productivity and income can lead to broader economic impacts. Moreover, dengue is a disease that disproportionately affects the poorest people in the country, who often live in precarious housing conditions and have limited access to healthcare and prevention [23].

In Latin America, factors such as the population’s immune profile and socioeconomic inequalities are related to the presence and magnitude of dengue. For Colombia, adjusted and annual estimates of dengue, stratified by the Concentration Index of Inequality, consistently showed a higher concentration of the disease among people in areas with limited resources and low socioeconomic status ^31^, in line with previous reports [24,25].

Just as dengue impacts populations differently, social dynamics also affect the spread of the disease. Migration to urban areas is linked to an increased risk of dengue transmission due to inadequate housing, poor sanitation, and limited access to healthcare services. In Colombia, the rapid urbanization process and migration to urban areas have been associated with a higher risk of dengue infection, particularly in low-income neighborhoods with poor infrastructure and sanitation has contributed to the expansion of dengue transmission and the emergence of new strains of the virus [26].

In this context, it is valuable to quantify the economic impact of dengue in Colombia. In this review, we collected and described all publicly available evidence on the economic burden of dengue in Colombia. We included 14 studies that showed a high financial burden on society, the healthcare system, households, and individuals. The costs associated with hospitalized and SD cases were much higher than ambulatory cases in all the studies. Direct costs ranged between USD 319 and USD 2048 for a case admitted to the hospital. The cost increased according to severity:USD 309 to USD 1108 for DWS and USD 1754 to USD 2048 for SD. For outpatient cases, the direct cost ranged from USD 40 to USD 135. The direct costs identified for Colombia in this SLR are consistent with estimates from published studies in other Latin American countries [27,28].

Additionally, it is important to note that although the health system in Colombia has high coverage, out-of-pocket expenses incurred during the diagnosis and recovery from dengue represented between 9% and 45% of the income, with transport to the care center as the main cost driver. These costs can be significant, especially for Colombia, where approximately 39,3% of the population lives in poverty [19].

Besides the direct costs, dengue significantly burdens society due to missed work and lost earnings as a results of death or disability [16]. The total costs incurred by the households (direct medical, direct non-medical and indirect costs) amounted to USD 86 million in 2010 compared to USD 20.8 million in 2011 and USD 25.8 million in 2012. One study calculated the lost income due to premature dengue deaths in USD 23.9, USD 3.9, and USD 3.9 million for 2010, 2011, and 2012, respectively [15]. Aggregated total costs vary according to incidence and mortality. In epidemic years, the aggregated total annual cost (direct and indirect) of dengue could be as high as USD 159.6 million, with ambulatory care and fatal cases representing 75% of the total cost [9].

The economic burden of dengue is not limited to the costs of treatment and lost income; the financial impact of surveillance and vector control programs must also be considered. Vector control measures typically include activities such as identification and elimination of breeding sites and fogging, requiring the purchase of insecticides and the deployment of trained personnel to conduct the activities. The estimated costs associated with the vector control program in Colombia ranged from USD 51.8 million to USD 59.3 million in an average year, showing a very high economic burden of dengue from the vector control program by the government/community [10]. This financial burden could be particularly challenging for low-income communities, which may lack the financial resources and infrastructure necessary to implement effective control measures. Governments and organizations may need to support these communities to ensure they have access to the resources necessary to control mosquito populations and reduce the incidence of dengue [29].

The introduction of a vaccine for dengue has the potential to reduce the economic burden of the disease. Vaccination could prevent dengue cases, reduce the need for costly treatments, hospitalizations, and deaths. Two studies included in this review showed that the average costs and loss of productivity per dengue episode were lower in the vaccinated group than in the not vaccinated group [13,17]. One of the studies did not include vaccination costs, which limits the validity of comparisons between groups. Nonetheless, if the vaccination costs are not high, the intervention could still be considered cost-effective. Thus, vaccination, as an additional tool within the integrated dengue control strategy—which should include vector control, personal protection, and an Information, Education, and Communication (IEC) strategy—could boost the economy by enabling individuals to remain productive and contribute to their communities. However, efforts must be made to ensure the vaccine is accessible and affordable for all who need it, particularly in high-incidence regions.

This review has some limitations. Economic burden calculations vary according to the dengue incidence, severity and mortality estimates and the transmission models used. This fact explains the broad differences between the costs reported in the studies. However, this problem is common to all studies that require estimating the number of dengue cases as the disease burden is frequently underestimated. Also, it is important to consider that surveillance systems are more sensitive to severe than non-complicated cases, potentially missing a substantial number of non-severe dengue cases in economic analysis. Another factor underlying the differences between studies is the medical services costs, which vary according to the healthcare center and region. In addition, the included economic studies estimated the costs of dengue by aggregating direct and indirect costs, based on willingness to pay for risk avoidance. However, these methods overlook important economic adjustment mechanisms. They fail to recognize that jobs do not remain vacant indefinitely because firms can hire new workers or invest in technology. In addition, these static approaches do not take into account population dynamics or changes in capital accumulation related to treatment costs. As a result, they often overestimate the economic burden of lost human capital, contributing to the inflated figures commonly seen in cost-of-illness and value-of-life analyses [30]. Nevertheless, the information presented here provides a useful summary of the economic burden of dengue in Colombia that could help guide policymakers in implementing dengue interventions.

## Conclusion

Dengue is hyperendemic in Colombia. The costs are substantial and significantly impact both individuals and society. The economic burden of dengue includes direct costs, such as medical expenses and indirect costs, such as lost productivity due to illness or death. The burden is exceptionally high among vulnerable populations such as children, the elderly, those living in poverty and regions with conditions that favor transmission and poor control of the disease.

Overall, addressing the socioeconomic impact of dengue in Colombia requires a concerted effort from all stakeholders, including the government, healthcare providers, and communities. Investment in prevention measures as part of an integrated strategy, such as vector control programs and vaccination, is crucial to reduce the impact of dengue on individuals and communities in Colombia.

## Supporting information

S1 PRISMA Checklist For studies about costs of dengue in Colombia. (DOCX)

S1 Text Search Strategies. (DOCX)

S2 Text Lists of the Excluded Publications and Reasons. (DOCX)

S1 Table Eligibility Criteria for Economic Burden Studies. (DOCX)

S2 Table Risk of Bias Assessment of Cost of Illness Studies. (DOCX)
