# Supplementary material for: Cost of dengue in Colombia: A systematic review
Source: PLoS Negl Trop Dis. 2024 Dec 12;18(12):e0012718. doi: 10.1371/journal.pntd.0012718 (PMC11670977; doi:10.1371/journal.pntd.0012718)
Supplement: S1 PRISMA Checklist — (DOCX) [file pntd.0012718.s001.docx]

**S1 Search Strategies**

**EMBASE search strategy**

| **Search line** | **Search terms** |
| --- | --- |
| **Disease** | |
| **1** | Dengue/ or severe dengue/ or dengue virus/ or dengue h?emorrhagic fever/ or dengue shock syndrome/ or dengue virus 1/ or dengue virus 2/ or dengue virus 3/ or dengue virus 4/ |
| **2** | (Dengue or severe dengue or dengue virus or dengue h?emorrhagic fever or alarm signs or DENV or DENV-1 or DENV-2 or DENV-3 or DENV-4).mp |
| **Epidemiology/clinical burden** | |
| **3** | Health care cost/ or cost of illness/ or hospitali#ation/ or hospital costs/ or hospital cost/ or economic aspect/ |
| **4** | (Economic aspect or financial aspect or cost of care or direct cost* or drug cost* or medical cost* or physician cost* or nurse cost* or clinic visit* or hospital visit* or cost* or average wage).mp |
| **5** | caregiver burden/ or caregiver/ or caregivers/ or medical leave/ or sick leave/ or health care financing/ or healthcare financing/ or health expenditures/ or drug cost/ or medical fee/ or fees,  medical/ or fees, pharmaceutical/ or hospital charge/ or work disability/ or absenteeism/ or productivity/ or medical leave/ |
| **6** | (economic burden or indirect cost* or productivity or societal burden or societal cost* or resource  utili#ation or resource u* or utilit* or workdays lost or school days lost).mp |
| **Combined results** | |
| **7** | 1 or 2 |
| **8** | 3 or 4 or 5 or 6 |
| **9** | 7 and 8 |
| **10** | 9 and (Colombia).mp |
| **11** | **Limits applied to total search result*** |

* Total citations in #10 limited to references published in English or Spanish from 2010 to 2020, and in human participants only.

**PubMed search strategy**

| **Search line** | **Search terms** |
| --- | --- |
| **Disease** | |
| **1** | dengue OR dengue virus OR dengue h?emorrhagic fever OR dengue fever OR dengue infection OR DENV OR DENV-1 OR DENV-2 OR DENV-3 OR DENV-4 |
| **Epidemiology/clinical burden** | |
| **2** | Economics OR cost OR cost analysis OR cost of illness OR healthcare cost OR cost-effectiveness OR direct cost OR economic cost OR disease cost OR absenteeism OR hospitali?ation cost OR accommodation cost OR transportation cost OR sick leave costs OR hospitali?ation OR  productivity OR productivity loss OR DALY |
| **Country** | |
| **3** | Colombia |
| **Combined results** | |
| **4** | 1 AND 2 AND 3 |
| **5** | **Limits applied to total search results*** |

*Total result in #4 limited to references published in English or Spanish from 2000 to 2020, and in human participants only.

**Grey literature sources**

| **Sources** | **Link** |
| --- | --- |
| WHO Library database (WHOLIS) | [Dengue and severe dengue (who.int)](https://www.who.int/health-topics/dengue-and-severe-dengue#tab%3Dtab_1) |
| Pan-American Health Organization (PAHO) | [PAHO/WHO \| Pan American Health Organization](https://www.paho.org/en) |
| Instituto Nacional de Salud:   - SIVIGILA IQEN (Informe Quincenal Epidemiológico Nacional) - BES (Boletin Epidemiologico Semanal) | http://www.ins.gov.co/Paginas/Inicio.aspx http://www.ins.gov.co/ |
| Sistema Integral de Información de la Protección Social (SISPRO) | <https://www.sispro.gov.co/Pages/Home.aspx> |
| Thesis catalogue of: | |
| ·         Universidad Industrial de Santander | |
| ·         Universidad de los Andes | |
| ·         Pontificia Universidad Javeriana | |
| ·         Universidad del Rosario | |
| ·         Universidad de Antioquia | |
| ·         Universidad del Valle | |
| ·         Universidad Nacional de Colombia | |

* Last 3 years (2017-2020) of conference abstracts will be searched
