# Supplementary material for: Cost of dengue in Colombia: A systematic review
Source: PLoS Negl Trop Dis. 2024 Dec 12;18(12):e0012718. doi: 10.1371/journal.pntd.0012718 (PMC11670977; doi:10.1371/journal.pntd.0012718)
Supplement: S1 Text — (DOCX) [file pntd.0012718.s002.docx]

**S2. Lists of the Excluded Publications and Reasons**

*Excluded for study design*

Alvis-Zakzuk, N. J., Castillo-Rodríguez, L., Diaz-Jimenez, D., Castañeda-Orjuela, C., Ochoa, M., & Cubillos, M. L. (2020). PNS31 Out-of-pocket health expenditures in Colombia: a systematic review. Value in Health, 23, S289.

Laserna, A., Barahona-Correa, J. E., Baquero, L., Castaneda-Cardona, C., & Rosselli, D. (2017, May). Economic impact of dengue in Latin America: a systematic review. In value in health (Vol. 20, No. 5, pp. A75-A76). 360 PARK AVE SOUTH, NEW YORK, NY 10010-1710 USA: ELSEVIER SCIENCE INC.

Rodriguez-Morales, A. J., Villamil-Gómez, W. E., & Franco-Paredes, C. (2016). The arboviral burden of disease caused by co-circulation and co-infection of dengue, chikungunya and Zika in the Americas. Travel medicine and infectious disease, 14(3), 177-179.

Salinas, M. A., Soto, V. E., & Prada, S. I. (2020). Análisis de costo-efectividad del uso del programa VECTOS en el control rutinario de enfermedades transmitidas por Aedes aegypti en dos municipios de Santander, Colombia. Biomédica, 40(2), 270.

Yasri, S., & Wiwanitkit, V. (2019). Costs of an Aedes aegypti vector control program. Cadernos de Saúde Pública, 35.

*Excluded for outcomes*

Abstracts From ISPOR Latin America 2019: Data and Value in Healthcare: 2020 and Beyond. Value in Health Regional Issues 2019"

Carabali, M., Lim, J. K., Palencia, D., Lee, K. S., Lee, J. S., Lozano, A., ... & Angel Villar, L. (2015, October). Dengue vaccine initiative project: burden of dengue fever in children and adults of piedecuesta santander, colombia. In american journal of tropical medicine and hygiene (Vol. 93, No. 4, pp. 429-430). 8000 westpark dr, ste 130, mclean, va 22101 usa: amer soc trop med & hygiene.

Coudeville, L., Baurin, N., Shepard, D., & Halasa, Y. (2016). Potential impact and economic value of dengue vaccination in 10 endemic countries. International Journal of Infectious Diseases, 45, 161.

del Campo, J. M., Morgan, G., Wilson-Barthe, M., Garcia, C., & Constenla, D. (2015, October). Cost of dengue vaccine introduction in the americas. In american journal of tropical medicine and hygiene (Vol. 93, No. 4, pp. 433-433). 8000 westpark dr, ste 130, mclean, va 22101 usa: amer soc trop med & hygiene.

Lee JS, Mogasale V, Lim JK, Carabali M, Lee KS, Sirivichayakul C, et al. A multi-country study of the economic burden of dengue fever: Vietnam, Thailand, and Colombia. PLoS Negl Trop Dis. 2017;11(10):e0006037."

Shepard, D. S., Halasa, Y. A., Zeng, W., Baurin, N., & Coudeville, L. (2017, November). Cost-effectiveness of dengue vaccination in five latin american countries. In american journal of tropical medicine and hygiene (Vol. 95, No. 5, pp. 567-567). 8000 westpark dr, ste 130, mclean, va 22101 usa: amer soc trop med & hygiene.

Yao, Y., Espana, G., & Perkins, A. (2018, January). Cost-effectiveness of dengvaxia vaccination of people with prior dengue virus exposure in ten latin american and asian countries. In american journal of tropical medicine and hygiene (Vol. 99, No. 4, pp. 508-509). 8000 westpark dr, ste 130, mclean, va 22101 usa: amer soc trop med & hygiene.
