# Supplementary material for: Cost of dengue in Colombia: A systematic review
Source: PLoS Negl Trop Dis. 2024 Dec 12;18(12):e0012718. doi: 10.1371/journal.pntd.0012718 (PMC11670977; doi:10.1371/journal.pntd.0012718)
Supplement: S2 Text — (DOCX) [file pntd.0012718.s003.docx]

**S1 Table. Eligibility Criteria for Economic Burden Studies**

|  | Inclusion criteria | Exclusion criteria |
| --- | --- | --- |
| Population | - Individuals of all ages with suspected or confirmed dengue in Colombia - Healthy population with potential previous exposure to dengue* | - Studies not reporting on patients with dengue or previous exposure to dengue - Studies conducted or reporting data outside of Colombia |
| Intervention | SLR not restricted by intervention criteria | |
| Comparison | SLR not restricted by comparison criteria | |
| Outcome* | - Cost of illness and economic burden of dengue, severe dengue and/or persistent dengue to patients and health services (according to payer type), and/or societal perspective, such as:   - Direct medical costs   - Direct non-medical costs   - Indirect societal costs - Vector control and surveillance costs - Productivity loss, such as:   - Sick leave or absenteeism   - Sick day lost due to illness (total, full, or partial)   - Disability-adjusted life years (DALYs)   - Years lived with disability (YLDs)   - Years of life lost (YLLs)   - Duration of illness prior to hospitalization   - Length of hospital stay   - Length of ICU stay   - Length of disease course   - Length of treatment | - Studies reporting none of the outcomes of interest |
| Study design | - Clinical trials:   - Randomized controlled trials (RCTs)   - Non-randomized controlled trials - Observational studies:   - Prospective and retrospective cohort studies   - Prospective and retrospective cross-sectional studies   - Retrospective case control studies   - Ecological studies or time-series analysis - Outbreak and surveillance reports - Systematic literature reviews if a meta-analysis is included - Genomic studies - Economic evaluations - Cost of illness studies | - Peer-reviewed publications that do not clearly outline methods and sources for data collection/analysis - In vitro studies - Clinical trials that do not report baseline/or analyse the placebo/control group in the results - News and opinion articles - Case reports - Narrative reviews, letters |
| Other criteria | | |
| Language | - English - Spanish | - Other languages |
| Study publication and data date | - Epidemiology: 2012 to 2020 - Cost: 2010 to 2020 | - Studies /data published outside the date limits:   - epidemiology: pre-2012   - cost: pre-2010 |

*Healthy population refers to individuals without dengue symptoms. This definition does not extend to other medical conditions.
