# Supplementary material for: Cost of dengue in Colombia: A systematic review
Source: PLoS Negl Trop Dis. 2024 Dec 12;18(12):e0012718. doi: 10.1371/journal.pntd.0012718 (PMC11670977; doi:10.1371/journal.pntd.0012718)
Supplement: S1 Table — (DOCX) [file pntd.0012718.s004.docx]

**S2 Table. Risk of Bias Assessment of Cost of Illness Studies**

| First author, year | Vásquez-Trujillo, 2020(1) | Shepard, 2016(2) | Castañeda-Orjuela, 2012(3) | Hernández, 2019(4) | Fitzpatrick, 2017(5) | Alfonso-Sierra, 2016(6) | Lee, 2017(7) |
| --- | --- | --- | --- | --- | --- | --- | --- |
| Analytical framework: what costs should have been measured? | | | | | | | |
| What was the motivation of the study? | To describe the economic burden of dengue epidemics between 2010 and 2016 in the State of Meta, Colombia. | To provide objective, systematic, comparable measures of dengue burden to track health progress, assess the application and financing of emerging preventive and control strategies, and, more generally, to inform evidence-based health policy. | To assess the burden of dengue disease in Colombia and its associated costs. | To assess the out-of-pocket expenses of the patients with a diagnosis of dengue in the cities of Medellín and  Montería, Colombia | To evaluate the cost (direct medical costs and control programme costs) and cost-effectiveness of sustained vector control, outbreak response and/ or medical case management, in the presence of a (hypothetical) highly targeted and low cost immunization strategy using a medium-efficacy vaccine. | To reports the cost analysis of these interventions. | To explore and understand socioeconomic factors affecting the level of disease severity that results in different levels of economic burden. |
| What was the perspective of the study? | Longitudinal perspective | Societal | Health care system | Patient | Societal | Health care provider | Decision maker |
| Was the appropriate epidemiologic approach taken? | Yes | Yes | Yes | Yes | Yes | NA | Yes |
| Was the study question well specified? | Yes | Yes | Yes | Yes | Yes | Yes | yes |
| Were all relevant, non-trivial cost components and their stakeholders identified? | Can't tell | Can't tell | No | Can't tell | Yes | No | Yes |
| Were necessary timeframes specified? | Yes | No | Yes | Yes | Yes | Yes | Yes |
| Was a case of disease or risk factor adequately and appropriately defined? | Yes | Yes | Yes | Yes | Yes | Yes | Yes |
| Was the counterfactual population occurrence plausible and meaningful? | NA | Yes | Yes | Yes | Yes | Yes | Yes |
| Methodology and data: how well were resource use and productivity losses measured? | | | | | | | |
| Was an appropriate method(s) of quantification used, such that additional, or excess, costs were measured? | No | Yes | No | Yes | Yes | No | Yes |
| Was an appropriate method(s) of quantification used, such that only costs specific to (caused by) the health problem were included (confounders controlled)? | Yes | Yes | Yes | Yes | Yes | Yes | Yes |
| Was an appropriate method(s) of quantification used, such that all important effects were captured? | Yes | Yes | Yes | NA | Yes | No | Yes |
| Was an appropriate method(s) of quantification used, such that important differences across subpopulations were accounted for? | Yes | Yes | No | No | Yes | NA | Yes |
| Was an appropriate method(s) of quantification used, such that the required level of detail could be provided? | Yes | Yes | Yes | No | Yes | No | Yes |
| Was the resource quantification method(s) well executed? | Yes | Yes | Yes | Yes | Yes | Yes | Yes |
| For population-based studies, were cost allocation methods, data and assumptions valid? | Can't tell | Yes | Yes | NA | Yes | Yes | Yes |
| For person-based studies, were appropriate statistical tests performed and reported? | NA | NA | NA | Yes | NA | NA | Yes |
| Were data representative of the study population? | Yes | Yes | Yes | Yes | Yes | Yes | Yes |
| Were there any other relevant resource quantification issues? | No | No | No | No | Yes | No | Can't tell |
| Were healthcare resources valued appropriately? | Yes | Yes | Yes | Yes | Yes | No | Yes |
| Was the approach for valuing production losses justified, and assumptions valid? | NA | Yes | NA | NA | Yes | NA | Yes |
| Was the inclusion of intangible costs appropriate | Can't tell | Can't tell | NA | Yes | Yes | NA | Yes |
| Was double counting of mortality-related production losses avoided? | NA | Can't tell | NA | NA | NA | NA | NA |
| Were losses valued appropriately, given the study’s perspective? | NA | Yes | NA | Yes | Yes | NA | Yes |
| Analysis and reporting | | | | | | | |
| Did the analysis address the study question? | Yes | Yes | Yes | Yes | Yes | Yes | Yes |
| Was a range of estimates presented? | Yes | Yes | Yes | Yes | Yes | No | Yes |
| Were the main uncertainties identified? | Can't tell | Yes | Yes | Yes | Yes | No | Can't tell |
| Was a sensitivity analysis performed on important (uncertain) parameter estimates? | No | Yes | Yes | No | Yes | No | No |
| Was a sensitivity analysis performed on key assumptions? (including the counterfactual) | No | Can't tell | Yes | No | Yes | No | No |
| Was a sensitivity analysis performed on point estimates? (based on confidence or credible intervals) | No | Yes | Yes | No | Yes | No | No |
| Was adequate documentation and justification given for cost components, data and sources, assumptions and methods? | No | Yes | Yes | No | Yes | Yes | Yes |
| Was uncertainty around the estimates and its implications adequately discussed? | Yes | Yes | Yes | Yes | Yes | Yes | Yes |
| Were important limitations discussed regarding the cost components, data, assumptions and methods? | Yes | Yes | Yes | Yes | Yes | Yes | Yes |
| Were the results presented at the appropriate level of detail to answer the study question (cost components; disease subtypes, severity, stage; subpopulation groups, cost bearers)? | Yes | Yes | Yes | Yes | Yes | Yes | No |
| Yes | 16 | 24 | 22 | 19 | 29 | 13 | 24 |
| NA | 5 | 1 | 5 | 4 | 2 | 7 | 1 |
| No | 6 | 2 | 4 | 7 | 0 | 11 | 4 |
| Can't tell answers | 4 | 4 | 0 | 1 | 0 | 0 | 2 |
| Quality score | 31 | 31 | 31 | 31 | 31 | 31 | 31 |
| Maximum score | 19 | 27 | 23 | 17 | 31 | 9 | 23 |
| Final score | 61,29% | 87,10% | 74,19% | 54,84% | 100,00% | 29,03% | 74,19% |

| First author, year | Salinas-  López, 2018(8) | Claypool, 2019(9) | El-Fezzazi, 2017(10) | Mora-Salamanca, 2020(11) | Castro-Rodríguez, 2015(12) | Castro-Rodríguez, 2016(13) | Zeng, 2018(14) |
| --- | --- | --- | --- | --- | --- | --- | --- |
| Analytical framework: what costs should have been measured? | | | | | | | |
| What was the motivation of the study? | To quantify the costs of vector-borne disease control programs at the local level in Colombia (2016) | To illustrate the bias from omitting intervention effects on other diseases, we developed single disease models of chikungunya and dengue and a combined model of the 2 diseases and used them to compare predictions of the incremental benefits and cost-effectiveness of an insecticide, a hypothetical chikungunya vaccine, a hypothetical dengue vaccine, and combinations of these interventions. | To compare breakthrough dengue disease–related resource utilization, direct, and indirect costs among participants receiving CYD-TDV with those receiving placebo during the 25 months of the active surveillance phase of two large-scale pivotal phase III studies | To estimate the burden of disease by Dengue, Chikungunya, and Zika in Colombia between 2013 and 2016. | To quantify the various components of the costs of a dengue case in Colombia | To present estimates from 2010 to 2012 for the burden of the disease and the overall cost, calculated as the sum of medical costs, income lost owing to premature death, loss of productivity, and expenditure on direct, indirect, and prevention and monitoring activities for dengue infection in Colombia. | To evaluate the cost-effectiveness of dengue vaccination in papulation similar to the trial sites on latin American and Asian countries. |
| What was the perspective of the study? | Policy maker | Health system | Societal | NR | Healthcare system and patient | Societal | Health system and societal |
| Was the appropriate epidemiologic approach taken? | Yes | Yes | Yes | Yes | Yes | Yes | Yes |
| Was the study question well specified? | Yes | Yes | Yes | Yes | Yes | Yes | Yes |
| Were all relevant, non-trivial cost components and their stakeholders identified? | Yes | Can't tell | Yes | NA | Yes | Yes | Yes |
| Were necessary timeframes specified? | Yes | Yes | No | Yes | Yes | Yes | Yes |
| Was a case of disease or risk factor adequately and appropriately defined? | Yes | Yes | Yes | Yes | Yes | Yes | Yes |
| Was the counterfactual population occurrence plausible and meaningful? | NA | Yes | Yes | NA | NA | NA | NA |
| Methodology and data: how well were resource use and productivity losses measured? | | | | | | | |
| Was an appropriate method(s) of quantification used, such that additional, or excess, costs were measured? | Yes | No | Yes | NA | Yes | Yes | Yes |
| Was an appropriate method(s) of quantification used, such that only costs specific to (caused by) the health problem were included (confounders controlled)? | Can't Tell | Yes | Yes | NA | Yes | Yes | Yes |
| Was an appropriate method(s) of quantification used, such that all important effects were captured? | Yes | Yes | Yes | Yes | Yes | Yes | Yes |
| Was an appropriate method(s) of quantification used, such that important differences across subpopulations were accounted for? | No | NA | Yes | Yes | Yes | Yes | No |
| Was an appropriate method(s) of quantification used, such that the required level of detail could be provided? | Yes | Yes | Yes | Yes | Yes | Yes | Yes |
| Was the resource quantification method(s) well executed? | Yes | Yes | Yes | NA | Yes | Yes | Yes |
| For population-based studies, were cost allocation methods, data and assumptions valid? | Yes | Yes | Yes | NA | Yes | Yes | Yes |
| For person-based studies, were appropriate statistical tests performed and reported? | Yes | NA | NA | Yes | NA | NA | NA |
| Were data representative of the study population? | Yes | Yes | Yes | Yes | Yes | Yes | Yes |
| Were there any other relevant resource quantification issues? | No | No | No | NA | No | No | No |
| Were healthcare resources valued appropriately? | NA | NA | Yes | NA | Yes | Yes | Yes |
| Was the approach for valuing production losses justified, and assumptions valid? | NA | Yes | Yes | NA | Yes | Yes | NA |
| Was the inclusion of intangible costs appropriate | Yes | NA | NA | NA | Yes | Yes | Yes |
| Was double counting of mortality-related production losses avoided? | NA | Yes | NA | Yes | Can't tell | Can't tell | Yes |
| Were losses valued appropriately, given the study’s perspective? | Yes | Yes | Yes | NA | Yes | Yes | Yes |
| Analysis and reporting | | | | | | | |
| Did the analysis address the study question? | Yes | Yes | Yes | Yes | Yes | Yes | Yes |
| Was a range of estimates presented? | No | Yes | No | yes | Yes | Yes | Yes |
| Were the main uncertainties identified? | Yes | Yes | No | yes | Yes | Yes | Yes |
| Was a sensitivity analysis performed on important (uncertain) parameter estimates? | No | Yes | No | yes | Yes | No | Yes |
| Was a sensitivity analysis performed on key assumptions? (including the counterfactual) | No | Yes | No | yes | Yes | Yes | Yes |
| Was a sensitivity analysis performed on point estimates? (based on confidence or credible intervals) | No | Yes | No | No | Yes | Yes | Yes |
| Was adequate documentation and justification given for cost components, data and sources, assumptions and methods? | No | Yes | Yes | NA | Yes | Yes | Yes |
| Was uncertainty around the estimates and its implications adequately discussed? | Yes | Yes | Yes | Yes | No | Yes | Yes |
| Were important limitations discussed regarding the cost components, data, assumptions and methods? | Yes | Yes | Yes | yes | Yes | Yes | Yes |
| Were the results presented at the appropriate level of detail to answer the study question (cost components; disease subtypes, severity, stage; subpopulation groups, cost bearers)? | Yes | Yes | Yes | yes | Yes | Yes | Yes |
| Yes | 19 | 24 | 21 | 18 | 26 | 26 | 26 |
| NA | 4 | 4 | 3 | 12 | 2 | 2 | 3 |
| No | 7 | 2 | 7 | 1 | 2 | 2 | 2 |
| Can't tell answers | 1 | 1 | 0 | 0 | 1 | 1 | 0 |
| Quality score | 31 | 31 | 31 | 31 | 31 | 31 | 31 |
| Maximum score | 17 | 27 | 17 | 29 | 27 | 27 | 27 |
| Final score | 54,84% | 87,10% | 54,84% | 93,55% | 87,10% | 87,10% | 87,10% |

**Referencias**

1. Vásquez-Trujillo A, Cardona-Arango D, Segura-Cardona AM, Parra-Henao GJ. Burden of dengue in the State of Meta, Colombia (2010-2016). Cad Saúde Pública. 2020 Jun 26;36:e00055119.

2. Shepard DS, Undurraga EA, Halasa YA, Stanaway JD. The global economic burden of dengue: a systematic analysis. Lancet Infect Dis. 2016 Aug;16(8):935–41.

3. Castañeda-Orjuela C, Díaz H, Alvis-Guzman N, Olarte A, Rodriguez H, Camargo G, et al. Burden of Disease and Economic Impact of Dengue and Severe Dengue in Colombia, 2011. Value in Health Regional Issues. 2012 Dec 1;1(2):123–8.

4. Hernández Sarmiento JM, Sánchez LMM, Romero EMD, Tabares MDR, Barreto ÁSB, Quintero NM, et al. Out-of-pocket of patients diagnosed with dengue in Medellin and Monteria, Colombia. Enf Infec Microbiol. 2020 Mar 13;39(1):7–11.

5. Fitzpatrick C, Haines A, Bangert M, Farlow A, Hemingway J, Velayudhan R. An economic evaluation of vector control in the age of a dengue vaccine. PLoS Negl Trop Dis. 2017 Aug;11(8):e0005785.

6. Alfonso-Sierra E, Basso C, Beltrán-Ayala E, Mitchell-Foster K, Quintero J, Cortés S, et al. Innovative dengue vector control interventions in Latin America: what do they cost? Pathog Glob Health. 2016;110(1):14–24.

7. Lee JS, Mogasale V, Lim JK, Carabali M, Lee KS, Sirivichayakul C, et al. A multi-country study of the economic burden of dengue fever: Vietnam, Thailand, and Colombia. PLOS Neglected Tropical Diseases. 2017 Oct 30;11(10):e0006037.

8. Salinas-López MA, Soto-Rojas VE, Ocampo CB. Costos de un programa de control del vector *Aedes aegypti* en municipios de Colombia: el caso de Girón y Guadalajara de Buga, 2016. Cad Saúde Pública. 2018 Nov 29;34:e00044518.

9. Claypool AL, Brandeau ML, Goldhaber-Fiebert JD. Quantifying Positive Health Externalities of Disease Control Interventions: Modeling Chikungunya and Dengue. Med Decis Making. 2019 Nov 1;39(8):1045–58.

10. El Fezzazi H, Branchu M, Carrasquilla G, Pitisuttithum P, Perroud AP, Frago C, et al. Resource use and costs of dengue: analysis of data from phase III efficacy studies of a tetravalent dengue vaccine. The American journal of tropical medicine and hygiene. 2017;97(6):1898.

11. Mora-Salamanca AF, Porras-Ramírez A, De la Hoz Restrepo FP. Estimating the burden of arboviral diseases in Colombia between 2013 and 2016. International Journal of Infectious Diseases. 2020 Aug 1;97:81–9.

12. Castro Rodríguez R, Galera-Gelvez K, López Yescas JG, Rueda-Gallardo JA. Costs of dengue to the health system and individuals in Colombia from 2010 to 2012. Am J Trop Med Hyg. 2015 Apr;92(4):709–14.

13. Castro Rodríguez R, Carrasquilla G, Porras A, Galera-Gelvez K, Yescas JGL, Rueda-Gallardo JA. The Burden of Dengue and the Financial Cost to Colombia, 2010–2012. Am J Trop Med Hyg. 2016 May 4;94(5):1065–72.

14. Zeng W, Halasa-Rappel YA, Baurin N, Coudeville L, Shepard DS. Cost-effectiveness of dengue vaccination in ten endemic countries. Vaccine. 2018;36(3):413–20.
